# Heterogeneity within Autism Spectrum Disorders: What have We Learned from Neuroimaging Studies?

**DOI:** 10.3389/fnhum.2013.00733

**Published:** 2013-10-30

**Authors:** Rhoshel K. Lenroot, Pui Ka Yeung

**Affiliations:** ^1^School of Psychiatry, University of New South Wales, Sydney, NSW, Australia; ^2^Neuroscience Research Australia, Sydney, NSW, Australia

**Keywords:** autism, psychiatry and developmental disabilities, intellectual disability, functional magnetic resonance imaging, structural magnetic resonance imaging

## Abstract

Autism spectrum disorders (ASD) display significant heterogeneity. Although most neuroimaging studies in ASD have been designed to identify commonalities among affected individuals, rather than differences, some studies have explored variation within ASD. There have been two general types of approaches used for this in the neuroimaging literature to date: comparison of subgroups within ASD, and analyses using dimensional measures to link clinical variation to brain differences. This review focuses on structural and functional magnetic resonance imaging studies that have used these approaches to begin to explore heterogeneity between individuals with ASD. Although this type of data is yet sparse, recognition is growing of the limitations of behaviorally defined categorical diagnoses for understanding neurobiology. Study designs that are more informative regarding the sources of heterogeneity in ASD have the potential to improve our understanding of the neurobiological processes underlying ASD.

## Introduction

The Autism Spectrum Disorders (ASD) are a group of lifelong neurodevelopmental syndromes which manifest in early childhood, defined by the presence of difficulties with social interactions and communication together with restricted, repetitive patterns of interests or behaviors (American Psychiatric Association, 2013). The variety of clinical presentations considered to fall within autism has gradually increased over the past 60 years. Leo Kanner first used the diagnosis in 1943 to describe a relatively homogenous group of individuals with deficits in all three domains but intact intellectual capacity (Kanner, 1968). Work by Lorna Wing and others then went on to place Kanner’s subgroup within a broader spectrum that shared some level of deficit in these core domains but was otherwise highly heterogeneous, including allowing a wider spread of cognitive function (Wing, 1981).

In the previous version of the Diagnostic and Statistical Manual IV (American Psychiatric Association, 2000), this heterogeneity was captured primarily through the different categorical diagnoses within the Pervasive Developmental Disorders (PDD). The PDD category included Autism, Asperger’s Syndrome, and Pervasive Developmental Disorder not otherwise specified (PDD-NOS), as well as two regressive neurodevelopmental disorders of early childhood frequently associated with autistic symptoms, Rett’s syndrome and Childhood Disintegrative Disorder. As research progressed, the clinical and neurobiological validity of the categorical distinctions between Autism, Asperger’s Syndrome, and PDD-NOS appeared increasingly doubtful, until they were dropped altogether in the recently released DSM-5 (American Psychiatric Association, 2013). Instead, a broader ASD diagnosis with dimensional specifiers of severity has been adopted, and a new diagnosis of Social Communication Disorder added for those individuals with problems in social communication but without the symptoms in the domain of restrictive and repetitive behaviors (RRIB) required for an ASD diagnosis.

An explicit goal of the recent reformulation of the diagnostic criteria for ASD was to refocus attention on the aspects of heterogeneity within ASD that were likely to be more meaningful than the previous categorical divisions, both clinically and in relation to underlying pathophysiology. This shift in conceptualization of ASD has occurred within a larger context of increasing dissatisfaction with existing diagnostic categories for a variety of psychiatric syndromes, particularly when seeking to link clinical symptoms to specific neurobiological processes (Insel et al., 2010; Lord and Jones, 2012; Uher and Rutter, 2012).

Heterogeneity of clinical presentation among individuals who meet criteria for a specific diagnosis is a particular impediment. One contributor to this heterogeneity is the checklist approach currently used to assign categorical diagnoses. While allowing a good level of reliability between diagnosticians, it has the unfortunate effect of allowing different individuals to meet the threshold for a particular syndrome without necessarily sharing many specific clinical features. A second contributor to heterogeneity of clinical presentations within a particular syndrome such as ASD is the extensive comorbidity between psychiatric diagnoses (Matson and Williams, 2013). It has been estimated that between 14 and 78% of children with ASD also meet criteria for Attention Deficit and Hyperactivity Disorder (ADHD) (Gadow et al., 2005; Gargaro et al., 2011), up to 42% for anxiety disorders (Simonoff et al., 2008; Matson and Williams, 2013), and between 25 and 70% have some level of intellectual disability (ID) (Fombonne, 2009). There are a wide variety of other symptoms not considered as “core” but which are nevertheless prominent in sizeable fractions of individuals with ASD, such as poor attention, seizure disorders, poor sleep, and gastro-intestinal dysfunction (Silver and Rapin, 2012). How these symptoms relate to the pathophysiology of ASD is not clear, but their frequency is suggestive that they may represent at least in part pleiotropic expressions of common processes (Brock, 2011; Rommelse et al., 2011).

In addition to the clinical heterogeneity within ASD, it is associated with a wide variety of different risk factors, implying the potential for many different pathways to generate symptoms (Geschwind, 2009; Herbert, 2010). ASD has been associated with over 100 different genes affecting different aspects of neural development and function (Betancur, 2011), as well as a wide variety of environmental factors (Herbert, 2010). Attempting to understand the links of specific risk factors to autism is further complicated by the different ways risk factors can relate to clinical phenomena, described as *equifinality* and *multifinality* (Cicchetti and Rogosch, 1996). Equifinality refers to the observation that a single clinical syndrome may be associated with many different risk factors. Multifinality describes the converse situation, where a single risk factor can be associated with different clinical outcomes. Examples pertinent to ASD include Fragile X, where up to 45% of affected individuals have been estimated meet criteria for ASD (but 55% do not) (Gallagher and Hallahan, 2012), and 22q11.2d syndrome, which appears to increase risk for ASD in some individuals (Antshel et al., 2007), and schizophrenia in others (Murphy et al., 1999). Research on genetic risk factors for ASD in general has been notable for the lack of specificity to ASD (Betancur, 2011). Risk-genes instead appear to confer vulnerability for a variety of neurodevelopmental disorders (Sahoo et al., 2011; Rapoport et al., 2012), suggesting that understanding the role of genetic risk factors for ASD will require identifying the factors that direct an individual with a risk-gene down the path to one neurodevelopmental disorder versus another as much as identifying the genes themselves (Moreno-De-Luca et al., 2013).

Investigators have taken different approaches to the clinical and neurobiological heterogeneity of ASD. Geschwind and Levitt (2007) drew attention to the variety of associated genetic syndromes, endorsing the view that the field should be considering a multiplicity of “autisms” rather than a single condition. They went on to hypothesize that what these “autisms” shared was a common abnormality in brain connectivity that occurred early in development and most strongly affected the links between frontal and temporal/parietal cortices. A large body of work has accrued using multiple neuroimaging techniques across a variety of presentations and ages that has shown impaired functional and structural connectivity between brain regions (Amaral et al., 2008; Horder and Murphy, 2012; Just et al., 2012; Travers et al., 2012).

However, limitations to this formulation have also been recognized (Vissers et al., 2012). While the evidence of abnormalities in connectivity are convincing, it does not in itself explain either how the observed abnormalities in connectivity explain the heterogeneity of specific presentations, or how they differentiate autism from a variety of other disorders characterized by poor connectivity between similar brain regions, such as ADHD and schizophrenia. Pelphrey et al. (2011) proposed an alternative approach, in which heterogeneity was constrained by narrowing attention to the deficits in social communication felt to be the heart of ASD, and focusing on early failures of the neural systems relevant to these. While acknowledging that disruptions in these systems could arise from many different sources, congruent with the observations of multiple risk factors, they argued that what was relevant to ASD was the convergence of these factors on the development of specific aspects of the social brain network, such as the posterior superior temporal sulcus (Pinkham et al., 2008; Kaiser et al., 2010).

Concerns have been raised that this approach focuses too closely on the social communication aspect of ASD, and thus not only does not address the range of clinically relevant symptoms, but also stands to lose information potentially crucial for determining what types of pathophysiology are relevant to a particular individual. Brock (2011) have argued that a better method is to consider the heterogeneity along with the findings: for example, rather than just determining whether the amygdala is hyper- or hypo-activated, one should characterize what differentiates those with hyperactivity from those with hypoactivity.

This leads to the strategy of tackling heterogeneity by identifying more homogeneous subgroups, with the hypothesis that this will decrease noise due to variation and facilitate detection of meaningful group differences or brain-behavior relationships (Volkmar et al., 2009). Comparing putative subgroups against each other can also test whether a particular feature distinguishing the subgroups is related to brain differences, or conversely, if there is something that appears consistently found in individuals meeting diagnostic criteria despite other heterogeneous aspects of the presentation. The subgroups explored the most to date are the different disorders within the PDD category in DSM-IV (American Psychiatric Association, 2000), but other ways of dividing ASD have also been used, including divisions based on clinical features such as regression or level of ID, or subgroups defined by presence of a specific risk factor such as gender or a known genetic disorder.

Heterogeneity can also be addressed by using a dimensional approach to relate variation in neuroimaging measures to some other aspect of the presentation (Constantino, 2011; Lord and Jones, 2012; Uher and Rutter, 2012). Being able to determine whether some aspect of the brain is predictive of a clinical symptom can both support the relevance of the imaging data, and perhaps, depending on how much is known about the function of those brain regions, inform the larger questions as to what neural processes are responsible for the clinical presentation.

We first briefly discuss which findings appear to be the most consistent in neuroimaging studies of ASD. The second section will review studies that have looked at subgroups within ASD, and the third section work that has instead focused on dimensional approach, both of the features considered as core symptoms of ASD and of common co-occurring conditions.

## What is Common within Neuroimaging Findings in ASD?

Most imaging studies of ASD done to date have been done taking the categorical diagnosis as their framework, asking the basic question of whether there are one or more brain differences corresponding to the clinical syndrome that can be recognized despite the various sources of heterogeneity. As is the case for other psychiatric disorders, clinical MRIs of individuals with autism do not typically show gross lesions or other abnormalities that can be used to distinguish affected individuals. Instead, alterations in brain structure or function are most easily detectable as differences between groups, and, given the variability of the syndrome, preferably using large samples.

Meta-analysis provides one method of combining data to create larger databases in which to search for patterns. A meta-analysis by Stanfield et al. (2008) included 43 structural neuroimaging studies comprised of data from over 800 subjects between 3 and 30 years of age with ASD and similar number of matched controls. Although there was considerable heterogeneity of results across the studies they included, significant findings on meta-analysis included enlarged total brain volume, hemispheres, cerebellum, and caudate in ASD, and decreased volumes in other brain regions including midbrain regions, regions of the cerebellar vermis, and area of the corpus callosum. Gray and white matter (WM) volumes were not reported separately in this meta-analysis.

Another recent meta-analysis of differences in brain morphometry in ASD limited its scope to fully automated voxel-based morphometry (VBM) studies of gray matter volumes (Via et al., 2011). Twenty studies met inclusion criteria, including 496 adolescents or adults with ASD (a combination of Autism and Asperger’s Syndrome) and 471 controls. Of the 17 studies that reported IQ, only one included subjects whose mean IQ was less than 70. This meta-analysis found no differences in global gray matter volume between ASD and controls; regional differences consisted of smaller gray matter volume in the ASD group in the amygdala-hippocampus complex and medial parietal regions. A linked meta-analysis by the same group of WM differences measured with VBM identified 13 eligible studies, including 246 patients with ASD, and 237 controls; while global WM volumes were not different, they did find evidence of increased volumes in regions relevant to language and social cognition (Radua et al., 2011).

Meta-analyses of functional magnetic resonance imaging (fMRI) studies have additional challenges due to the even greater variety of possible tasks and conditions to be compared. Philip et al. (2012) examined available fMRI studies in ASD across six different functional domains: visual processing, executive function and language tasks, basic social processing, and more complex challenges of social cognition. They performed their meta-analysis using the activation likelihood estimation (ALE) method, in which data regarding loci of activations are spatially normalized and then the overlap calculated across different experiments (Eickhoff et al., 2009). Ninety papers met inclusion criteria, with an average sample size of ASD participants of 12. Although the wide variety of tasks used made comparison challenging, there were regions of activation that were significantly different between the ASD subjects and controls in each of the functional domains examined. Notable findings included a tendency for decreased activation in ASD across several prefrontal and subcortical brain regions during tasks tapping executive function. Activation patterns in the superior temporal gyri were significantly different between ASD and controls across several domains, although the direction varied: ASD had decreased activation during tasks related to auditory and language processing, but increased during tasks of simple social processing, and mixed findings as demands became more complex. The authors also provided a qualitative review of available studies of functional connectivity that could not be included in an ALE analysis, finding multiple observations of decreased connectivity between areas of the cortex in ASD across a variety of resting or task based paradigms, and conversely some instead of increased connectivity, particularly between subcortical and cortical areas.

Robustness of findings in imaging studies has been limited by the small sample sizes and methodological variability of the studies available. However, technical advances in methods for data acquisition and automated processing are making multi-site large-scale imaging studies increasingly feasible. One such collaboration is the Autism Brain Imaging Data Exchange (ABIDE^1^), a consortium of investigators in which members contribute both published and unpublished resting-state fMRI data from ASD subjects and controls obtained using similar clinical and imaging protocols. The first paper from this consortium reported on measures of brain connectivity in a dataset collected across 17 sites. Neuroimaging data included in this analysis were limited to that collected from males (360 ASD, 403 controls) who had IQ within 2 standard deviations of the overall sample mean of 108 (Di Martino et al., 2013). The results helped to clarify conflicting results from earlier studies, confirming that both hyper- and hypo-connectivity are characteristic of brain activity in ASD. Hypo-connectivity was much more prominent, affecting to varying degrees all cortico-cortical connections tested, with particularly strong effects in unimodal association areas such as the fusiform and superior temporal gyri, paralimbic regions such as the insula and paracingulate cortex, and connections between hemispheres. Hyperconnectivity was more limited, affecting primarily subcortical nuclei and parietal cortex. The study represented a significant advance in reconciling previously inconsistent observations (Muller et al., 2011; Vissers et al., 2012), although the population studied was limited to the subset of individuals with ASD with normal range IQ, and did not have the younger subjects needed to answer questions around early developmental changes.

In summary, despite the clinical and etiological heterogeneity with ASD, the use of quantitative methods to look for common patterns across datasets collected thus far has detected evidence of some relatively consistent differences in both brain structure and function on a group level. However, a significant contributor to differences in findings among studies of ASD is age.

### Age effects

As suggested by the meta-analyses above, structural neuroimaging studies of adolescents and adults have had inconsistent findings, with some reporting enlarged volumes (Piven et al., 1995), but the majority normal or even reduced volumes (Garber et al., 1989), consistent with the meta-analyses of VBM data in older individuals by Via et al. (2011) discussed above (Radua et al., 2011). In contrast, studies of head circumference and brain volumes in children with autism have suggested that brain enlargement is a more consistent finding (Wolff, 2013). In the meta-analysis by Stanfield discussed above, age effects are described for the amygdala, such that the differences in amygdala volume correlated with age, becoming larger in younger subjects. While this analysis did not find similar age effects for total brain volume, unlike what had been reported in a previous meta-analysis (Redcay and Courchesne, 2005), there are increased effect sizes for larger total brain volumes in younger children. These and similar observations have led to the hypothesis that the trajectory of brain development in some individuals with ASD may show a two-step deviation: an early acceleration in brain growth, followed by a flattening of the growth trajectory or even relative loss in brain volume sometime during early adolescence (Aylward et al., 2002; Redcay and Courchesne, 2005).

It should be noted that while increased early brain volume is one of the most consistently reported observations, it has not been found by all studies (Raznahan et al., 2013a). A recent paper re-examining past reports of increased head circumference in ASD found evidence to suggest that much of this could actually due to bias from cohort effects within commonly used population databases such as the CDC. Those studies in which control data had been collected from the same community as the ASD group were less likely to find head circumference differences (Raznahan et al., 2013b). MRI comparisons of global gray and WM volumes in young children have also had mixed results. Studies that limited participants to those that met narrow DSM-IV criteria for Autism were less likely to find group differences, while larger brain volumes were more frequently observed when the patient group was extended to the broader autism spectrum. A recent study of brain volumes in toddlers with regressive versus non-regressive autism (Rogers, 2004; Hansen et al., 2008) of the same clinical severity also only found increased brain volumes in males with the regressive subtypes, while males with non-regressive autism were not different than controls. Brain volumes were not increased in females with either regressive or non-regressive autism, although sample size differences may have affected the ability to detect differences in the latter, as there were many more males in the study than females (114 males/22 females) (Nordahl et al., 2011). These findings, together with the observation that many genetic disorders associated with autism often result in microcephaly (Betancur, 2011), suggest that early increased brain growth is present in only a subset of individuals with ASD (Raznahan et al., 2013a).

There is as yet relatively little work attempting to assess developmental shifts in brain functional activity in ASD. In a recent meta-analysis, Dickstein et al. (2013) addressed developmental questions in fMRI results by using ALE methods to directly compare fMRI study results in children less than 18 years old with those obtained in adults. Tasks were split into those testing aspects of social function, such as theory of mind, face processing, and language, versus non-social capacities such as executive function and reward processing. Forty-two studies met inclusion criteria: 18 studies in children (including 262 ASD participants, average age 12.95 ± 1.74 years) and 24 in adults (288 participants, average age 30.55 ± 4.94 years), with similar numbers of age-matched controls. They reported that loci of both hyperactivation and hypoactivation were more pronounced in the younger subjects: in the group of social tasks, the convergence of hyperactivation was higher in the children in the left postcentral gyrus, and hypoactivation greater in the right parahippocampal gyrus/hippocampus and right superior temporal gyrus. In the non-social tasks, hyperactivation was greater in the ASD children in areas such as the right insula, right middle frontal gyrus, and left cingulate gyrus, while hypoactivation was more pronounced in the right middle frontal gyrus; there were no instances in either condition where convergences of hypo- or hyperactivation were greater in the adult ASD group. While the results need to be followed up in longitudinal analysis, they do suggest that development stage plays an important role in functional differences in ASD as well.

## Subgroups within ASD

### Subgroups defined by clinical features

#### Autism and Asperger’s syndrome

As discussed above, until the recent revision of DSM-5, ASD were divided into several categorical diagnoses. The question of whether Autism and Asperger’s syndrome should be considered different disorders has been contentious, particularly when comparing individuals with Asperger’s syndrome to those with Autism and normal cognitive function (Kozlowski et al., 2012; Planche and Lemonnier, 2012). Studies directly comparing the two have tended to report evidence of more severe brain abnormalities in Autism, with Asperger’s syndrome being intermediate (Lotspeich et al., 2004; Schumann et al., 2004). The recent meta-analysis of VBM measures of gray matter volume described above (Via et al., 2011) did not find evidence of significant differences in gray matter volume between Autism and Asperger’s syndrome, supporting the hypothesis that the conditions had similar neural substrates with differing levels of severity.

#### Intellectual disability

It is estimated that approximately of 60–80% of the total ASD population have mild to severe ID (Fombonne, 2003). How best to understand the relationship of ID to the core features of ASD is not clear – whether they should be considered as arising from the same fundamental process in individuals with both, whether ID should instead be treated as a co-occurring disorder, or whether ID may serve as an “unmasking” element that decreases an individual’s ability to compensate for other factors that place them at risk for autistic behaviors (Skuse, 2007).

A handful of studies have compared ASD with and without co-occurring ID, often referred to as “low-functioning autism” (LFA), and “high-functioning autism” (HFA). A study of global brain volumes and amygdala and hippocampal volumes in a group of children and a group of adolescents, divided into the four subgroups of LFA and HFA, Asperger’s syndrome and matched controls, did not find significant differences in patterns of neural abnormalities between LFA and HFA; both had significantly enlarged amygdala and hippocampal volumes in the younger children but not adolescents, and global brain volumes were the same as controls throughout (Schumann et al., 2004). A cross-sectional analysis of a subset of the same sample reported differences in cortical folding patterns between the four groups (Nordahl et al., 2007). Cortical morphometry and sulcal depths were modeled using a surface-based registration system (Van Essen et al., 2001). The LFA group had an area of deeper sulci than controls in the left frontal operculum and anterior insula; in the HFA group, sulcal depth was deeper in the left parietal operculum, approximately 12 mm distant from the area affected in the LFA group, and correlated with a similarly affected region in the right hemisphere. In the Asperger’s syndrome group, this region was not affected, but there was evidence of greater sulcal depth bilaterally in the intraparietal sulcus. Shape analysis showed an abnormal region in the pars opercularis portion of the left inferior frontal gyrus of the LFA group, which coincided with the sulcal depth abnormality. The sample was also divided into a younger (7.5–12.5 years) and older (12.75–18.5 years) in order to explore developmental effects: similarly to the study of amygdala size in this cohort, findings were more pronounced in the younger group, despite the smaller sample size, and no longer evident in the adolescent group.

Scanning children with intellectual disabilities is challenging, and relatively few studies have focused on LFA. Riva et al. (2011) compared brain volumes in a group of children with ASD aged 3–10 years, average IQ of approximately 52, against controls with normal IQ. They reported similar global brain volumes between the two groups, but a pattern of regional gray matter deficits in the autistic group. Other studies have used control groups matched on level of ID. Predescu et al. (2010) did not find differences between 15 children aged 2–8 with ASD and 10 age-matched children with developmental delay (DD) in global gray and WM volumes measured using VBM. A slightly larger study of 34 children aged 2–7 years with ASD and 13 controls matched for age and developmental level also did not find any significant differences in brain volumes, although there appeared to be a positive relationship between developmental stage in the developmentally delayed group that was absent in the ASD children (Zeegers et al., 2009). A study in older children with significant DD (27 ASD, 17 controls; both groups had chronologic age of approximately 14 years and developmental age of approximately 4.5 years) reported that the area of the corpus callosum was significantly smaller in the ASD group, although head circumference and cerebellar volumes were not different (Manes et al., 1999). A different approach was taken by Hrdlicka et al. (2005), who used cluster analysis to determine which traits grouped together across several domains, including brain volumes, autistic symptoms, IQ, facial dysmorphism, and comorbidities such as epilepsy. They found that level of ID differentiated between clusters, while ASD symptoms did not; more severe ID was associated with smaller volumes of the amygdala, hippocampus, and corpus callosum genu/splenium, along with higher frequency of epilepsy, facial dysmorphic features, and abnormal early psychomotor development. A significant limitation to the studies available thus far is their relatively small size. Compounding this is that the pathology underlying ID and DD is itself not well understood, and so control groups defined on basis of cognitive function are likely to introduce additional variation related to the causes of the cognitive impairment.

### Subgroups defined by risk factors

#### Environmental risk factors

Another strategy for parsing heterogeneity is subdividing based on the presence of a specific risk factor, either comparing individuals that have a specific risk factor who also have ASD features against those who do not, or comparing individuals with ASD and a specific risk factor against idiopathic ASD (iASD) and controls in order to see if the same patterns of differences is present regardless of the presence of the risk factor. Analyses of this type thus far have been carried out in primarily in regards to genetic risks. Although ASD has also been associated with a variety of environmental risk factors (Herbert, 2010), much less is yet known about how these might relate to brain differences. The strongest environmental risk identified thus far is severe early neglect, which has been associated with development of autistic behavioral features (Rutter et al., 2007), although studies of children raised in these conditions have also shown a capacity for significant improvements on exposure to a socially enriched environment that ASD generally does not. Some small neuroimaging studies have been done in these populations, which have documented decreased brain volumes and abnormal WM architecture, but the relationship of brain findings to autistic symptoms in these subjects is not known (Bos et al., 2011).

Prenatal exposure to maternal autoantibodies has been suggested as another environmental risk factor potentially playing a role in some individuals with ASD. The blood-brain barrier is permeable to maternal IgG during prenatal development, and maternal autoantibodies have been observed to react with fetal brain tissue, with reactivity to several specific antigens in the 37 and 73 kDa range of molecular weight linked to significantly increased risk for ASD in offspring (Braunschweig et al., 2013). Prenatal exposure of rhesus macaques to these autoantibodies from mothers of ASD children resulted in abnormal social function and a more rapid increase in brain volume in the males (although not females) during the first two years of life compared to controls (Bauman et al., 2013). These intriguing findings were followed up by a study of maternal autoantibodies and brain volume in male children with ASD and matched controls (Nordahl et al., 2013). In this study, 7.5% of the mothers of ASD children and none of the mothers of controls had the autoantibodies in the 37/73 kDa range; and the children of this subset had significantly larger brain volumes than the children with ASD who did not have exposure to these autoantibodies. Together, these findings support the hypothesis that there may be a subgroup of individuals with ASD in which pathophysiology is linked to a particular immune-mediated process during prenatal development. There have not been imaging studies yet related to other possible environmental factors such as prenatal maternal influenza.

#### Genetic risk factors

As extensively reviewed elsewhere, ASD is highly heritable (Ronald and Hoekstra, 2011), and has been linked to a large number of genetic risk factors (Betancur, 2011). The Y chromosome could be considered one of the strongest of these, conferring up to a 4:1 greater risk in males compared to females (Fombonne, 2009). Otherwise, the genes identified to date with the strongest impact on risk for ASD are those associated with known genetic neurodevelopmental disorders which include an increased likelihood of ASD (Fombonne, 2009). Examples include Fragile X syndrome (FXS) (Gallagher and Hallahan, 2012) and 22q11.2d, also known as velo-cardio-facial syndrome (VCFS) (Antshel et al., 2007). However, even in these disorders, ASD symptoms are only present in a subset of affected individuals, and each of these syndromes increases risk for a number of psychiatric disorders. Neuroimaging has begun to be used to try to determine whether having the ASD phenotype or not in individuals with the same genetic abnormality is reflected in variation in brain structure, and also whether brain differences associated with the ASD phenotype in a specific genetic condition are similar to those in “idiopathic” ASD. Such studies within specific genetic disorders have the additional benefit of allowing comparison of individuals with ASD and ID within a relatively homogeneous cohort. There is an element of controversy regarding the relationship of ASD symptoms to genetic disorders such as FRX, with some arguing that despite appropriate scores on standardized assessments, a closer analysis of clinical features of individuals with genetic syndromes reveals that similarities are superficial, and symptom profiles are not the same as in individuals with iAUT (Moss and Howlin, 2009). An alternative is that the pattern appears atypical precisely because they represent a subgroup associated with a single major genetic risk factor, and so do not display the same characteristics described as averaged observations from a much more heterogeneous group.

#### Gender

Autism spectrum disorders is significantly more common in males than females (1 in 54 in boys and 1 in 252 in girls, up to 5 times more frequent in boys; Center for Disease Control and Prevention, 2012), although there has been longstanding debate whether the risk factors disproportionately affects males, or if instead other protective factors tend to make symptoms less noticeable in girls. It has also been uncertain whether the proportion of males and females may change based on the level of ID. Several early studies reported an interaction of gender with ID, such that the ratio of males to females is nearly equal in individuals with ID, and becomes more prominent in the groups with higher IQ (Wing, 1981; Lord et al., 1982; Volkmar et al., 1993). However, some more recent studies have not found a relationship of gender ratio and IQ (Carter et al., 2007; Hartley and Sikora, 2009; Mandy et al., 2012). The preponderance of males in recruitment samples has resulted in many imaging studies excluding females altogether, in order to reduce potential variance related to gender. Those that have included both males and females have generally had insufficient numbers of females to examine effects of gender on outcomes, and there have only been a few MRI studies that have addressed differences in gender directly.

Most studies thus far that have reported on gender effects have not found significant differences in which brain areas are affected, although the magnitude of effects in areas has tended to be larger in females (Bloss and Courchesne, 2007; Schumann et al., 2009, 2010; Calderoni et al., 2012). One study however found reduced cerebral GM and WM volumes and reduced temporal GM volumes in females versus males with ASD; in addition, cerebral, frontal, parietal, and occipital WM volumes were only correlated with age in girls but not in boys (Bloss and Courchesne, 2007). This was consistent with the gender effects observed in a meta-analysis of brain structural differences in ASD, which found that differences in the cerebellum were more likely to be observed when there were fewer males included in the study, suggesting that females may be contributing greater differences (Stanfield et al., 2008).

A notable recent study, the largest to date designed explicitly to examine gender differences, found that brain regions affected in ASD males had little overlap with those affected in ASD females (Lai et al., 2013a). In this study, VBM was used to compare gray and WM global and regional volumes in high-functioning adult males and females with ASD (age range 18–49; 30 males with ASD; 30 females with ASD; 30 male controls and 30 female controls). There were not significant interactions of gender and diagnosis for gray matter volumes. However, for WM, several regions had divergent findings for males and females. In the temporo-parieto-occipital region, females with ASD had larger WM volumes than female controls, while there was no difference in males; while WM in the internal capsule in the area around the basal ganglia was larger in the ASD males than male controls, but smaller in ASD females than female controls. In addition to the difference in direction of findings, there was little spatial overlap between affected regions in males and in females. These findings suggest a difference in neuroanatomical substrates of ASD for males and females, despite similar clinical characteristics. Although in general females who have been identified with ASD have tended to be characterized as clinically more severely affected than males (Dworzynski et al., 2012), suggested by some as due to ascertainment biases, this study as well as the others described above did not find gender differences in their samples in either ID or symptom severity (Bloss and Courchesne, 2007).

There has been one fMRI study to date that targeted gender differences, comprised of a verbal fluency task and a mental rotation task (Beacher et al., 2012). These tasks were chosen based on previous evidence of gender specific performance in health populations: females tend to perform better than males on measures of verbal fluency (Herlitz et al., 1997), and males better than females on tests of mental rotation (Crucian and Berenbaum, 1998; Astur et al., 2004; Parsons et al., 2004; Kozaki and Yasukouchi, 2009). The authors found evidence of interaction of group and gender. On the verbal fluency task, AS males, but not females, had greater activation in the left medial superior frontal gyrus than controls. On the mental rotation task, AS males had greater activation in the left precuneus, bilateral occipital gyri, and left inferior temporal gyrus than controls; the opposite was true for females, with control females having greater activation in the same regions. The authors speculated that differences could have been due to gender differences in cognitive styles.

#### Genetic syndrome

Fragile X Syndrome is an X-linked disorder, the most common inherited form of ID, caused by a trinucleotide repeat in the Fragile X mental retardation 1 (FMR1) gene (Gallagher and Hallahan, 2012). It is estimated that 25–47% of individuals with FXS have ASD (Gallagher and Hallahan, 2012), resulting in 2–6% of all ASD cases (Reddy, 2005; Hagerman et al., 2010). Kaufmann et al. (2003) were the first to directly compare children with FXS and ASD, finding hypoplasia of the posterosuperior vermis in both groups compared to controls. A study of 10 adults with FXS, 10 iASD, and 10 TD using VBM also reported decreased volume of the cerebellar vermis in FXS and iASD compared to TD; the FXS group had increased volumes of caudate and dorsolateral prefrontal cortex (PFC), and decreased volumes of left postcentral, middle temporal, and right fusiform gyrus (FG) compared to both ASD and TD (Wilson et al., 2009). Meguid et al. (2010) reported on a comparison of cortical thickness in 10 children with iAUT and 7 children with FXS + AUT; they found that that for the most part there were no significant differences in measures of cortical thickness, gyrification, or sulcal depth between the two groups, except that the iAUT had thinner cortex in the left medial frontal and anterior cingulate cortices, which correlated with an index of social maturity.

A series of reports using different imaging analysis methods have come from work by Hazlett and colleagues regarding a longitudinal study of boys with FXS recruited between 2–4 years of age, including a group who had FXS with autism (FXS + AUT), FXS without autism (FXS-AUT), iASD, and controls (TD, a mix of typically developing and developmentally delayed children). In the baseline study using predefined regions of interest they found that the FXS group, both with and without autism, had enlarged caudate and decreased amygdala volumes, compared to both TD and iAUT. The most significant finding in the iAUT group was enlarged amygdala volume compared to either FXS or TD (Hazlett et al., 2009). At the next time point, when subjects were 5–6 years of age, additional differences were observed. Global volumes were similar between the FXS and iAUT groups, in both cases larger than the TD group, but frontal lobe gray and WM volumes were smaller in the FXS than in iAUT, and temporal WM and cerebellar volumes were larger (Hazlett et al., 2012). A longitudinal analysis of the same cohort using VBM found a complex pattern of differences between the groups. Interestingly, several regions important for social function such as medial PFC, orbitofrontal cortex, superior temporal sulcus, and temporoparietal region appeared to differ in opposite directions, being smaller in the FXS (including FXS + AUT) and larger in the iAUT. There was no significant difference in the overall severity of the autistic symptoms, which the authors interpreted as an example of different patterns of brain structural differences underlying similar symptoms (Hoeft et al., 2011).

Rett’s syndrome (RS) is an X-linked genetic disorder commonly associated with autistic features that was previously included within the same PDD category as ASD. Most affected individuals have a mutation in the Methyl-CpG-binding Protein 2 (MECP2), a transcription regulator important in activity-dependent synaptic maturation (Amir et al., 1999). As evidence grows supporting the role of synaptic development as a potential convergent pathway for pathophysiology in ASD, there has been strong interest in the MECP2 mutation as a prototypic model (Neul, 2012). Imaging studies of RS have described decreases in both gray and WM volume affecting frontal, temporal, and parietal regions (Naidu et al., 2001). Decreased volumes are consistent with the characteristic microcephaly, and appear to be more pronounced in individuals with more severe clinical phenotypes (Carter et al., 2008a). Interestingly, a study of the milder preserved speech variant subtype of RS found that while 76% of a cohort of 17 intermediate and high-functioning participants met criteria for ASD, only 3 had microcephaly, 11 had normal head circumference, and 2 were even macrocephalic (Zappella et al., 2001). There have not been studies to date explicitly examining imaging findings in RS in relationship to the autistic phenotype.

Velo-cardio-facial syndrome is caused by a deletion in the 22q11.2 region, and associated with increased risk of ASD (Antshel et al., 2007), as well as schizophrenia and other psychiatric conditions, and DDs (Shprintzen, 2008). One study to date has compared brain structures in VCFS with ASD to those without ASD. This study found that those with an ASD diagnosis had larger right amygdala volume (Antshel et al., 2007), consistent with other reports of increased amygdala size in ASD.

Down’s syndrome is the most common genetic cause of ID, and has been reported as being comorbid with ASD in a subset of between 1–11% (Lowenthal et al., 2007). Brain volumes in DS subjects are usually reported as smaller than controls. Findings have been mixed when comparing DS with or without autistic features. Studies that further subdivide DS by presence of autistic features have not found differences total brain volume or total cerebellar volume between those with (DS + AUT, ID + AUT) or without (DS-AUT, ID-AUT) autistic features (Kaufmann et al., 2003; Spencer et al., 2006; Carter et al., 2008b). Some evidence of differences however has been identified in cortical areas related to social functions such as the thalamus and left superior temporal sulcus (Spencer et al., 2006), and associations with motor or RRB in brainstem and cerebellar WM (Kaufmann et al., 2003; Carter et al., 2008b).

#### Other genetic risk factors

A large number of other genes not associated with specific genetic disorders have also been linked to increased risk for ASD. Most studies of the effects of these genes on brain structure in relation to ASD thus far have focused on demonstration of the effects of risk-genes on relevant aspects of brain structure or function within healthy subjects (e.g., contactin-associated protein-like 2 (CNTNAP2) (Scott-Van Zeeland et al., 2010a; Tan et al., 2010; Dennis et al., 2011; Whalley et al., 2011); homeobox A1 (HOXA1) (Canu et al., 2009; Raznahan et al., 2012); MET receptor tyrosine kinase (MET) (Hedrick et al., 2012), oxytocin receptor (OXT) (Inoue et al., 2010), and brain-derived neurotrophic factor (BDNF) (Raznahan et al., 2009). However, a few have sought to determine if risk alleles contribute to heterogeneity within ASD and may be useful for intra-diagnostic stratification; i.e., whether ASD subjects with a specific risk-gene allele have differences in brain structure or function from ASD subjects who do not have that particular allele.

Monoamine-oxidase A (MAOA) is an enzyme found in the brain that is a key regulator of serotonin, dopamine, and norepinephrine, all neurotransmitters that have been linked with ASD. VNTR, a polymorphism in the promoter region for the MAOA gene, has been demonstrated to affect the level of activity of this enzyme. A large body of work has linked the low activity (LA) allele to a variety of adverse effects on cognition and behavior, including decreased IQ and worse symptomatology within ASD (Cohen et al., 2003). A study of the relationship of MAOA and ASD compared brain volumes between individuals with the high (HA) versus LA alleles of VNTR, both within a sample of young males with ASD (18–35 months of age, 17 HA, 12 LA) and controls (7–18 years of age, 28 HA, 11 LA). While there was no effect of the allele type in the control subjects, within the ASD sample the LA allele was associated with larger volumes of both gray and WM (Davis et al., 2008).

MET is another candidate genetic risk factor with relatively robust support for a role in ASD. MET is a gene encoding a protein within the ERK/PI3 signaling pathway, and is closely regulated during the development of excitatory neurons during synapse formation in regions of the brain important for social cognition (Levitt and Campbell, 2009). Variations in MET have been linked to increased risk for ASD (Campbell et al., 2010), and in animal models have been associated with developmental abnormalities consistent with ASD phenotypes (Judson et al., 2009). Alleles of rs158830, located within the promoter region of MET, have been of particular interest because of their effects on transcription and protein expression of MET. The presence of the “C” allele of rs158830 has been associated with more severe deficits in social function and communication (Campbell et al., 2010). Rudie et al. (2012) measured the impact of the rs158830 risk allele on structural and functional brain development in a population of 162 children and adolescents with (*n* = 75) and without (*n* = 87) ASD. Participants contributed to one or more of three separate neuroimaging experiments: an fMRI paradigm involving passive viewing emotional faces; a resting fMRI scan; and a diffusion tensor imaging scan. Group differences between ASD and controls were present for both functional imaging paradigms. Independent of diagnosis, the MET risk allele was also associated with abnormalities in brain activation in both paradigms, and with decreased fractional inositropy in several WM tracts in related regions. Notably, the effects of the risk allele were more pronounced in the ASD group than in the controls. Across all three testing conditions, the intermediate heterozygote (GC) within the ASD group were more similar to the high risk homozygote (CC); while within the control group the heterozygote condition was more similar to the low risk (GG) homozygote. The differences between the ASD participants with and without the risk allele supported the value of stratifying samples both by diagnosis and by specific genetic risk factors.

## Dimensional Approaches to Heterogeneity

Although most imaging studies in ASD have concentrated on group comparisons, there have been some which have sought instead to determine whether the variation observed in clinical characteristics could be linked to differences in brain structure and function. Some of these have concerned the different domains considered as part of the core criteria of ASD, while others have started to explore the impact of common co-existing features such as ID, anxiety symptoms, and problems with attention and impulsivity. As the vast majority of the imaging literature is based on DSM-IV criteria, this review will accordingly consider the domains of language, social communication, and repetitive/restricted interests and behaviors separately, rather than following the revised criteria in which language and social interaction are combined (American Psychiatric Association, 2013).

### Social cognition

Impairment in social interactions is the defining feature of ASD. There has been significant progress in delineating the neural systems playing key roles in the enormously complex cognitive processes underlying routine social activity, highlighting brain structures such as the medial frontal and superior temporal cortex, insula, cingulate, and limbic regions (Blakemore, 2008). Neuroimaging studies comparing ASD with controls have demonstrated that these regions among those most consistently showing abnormalities, confirming their likely involvement in the clinical phenomena (Di Martino et al., 2009; Sugranyes et al., 2011).

However, as in other aspects of ASD, the severity of impairment of social function can vary widely. Fewer studies have attempted to determine if MRI measures of these neural systems predicts the clinical symptoms. The amygdala has been one of the regions that has received the most attention, due to its well-established role in relevant aspects of social cognition such as response to facial expressions and threat detection (Adolphs et al., 2005; Adolphs, 2010; Pessoa, 2010), and evidence of abnormal structure and function of the amygdala in ASD (Abell et al., 1999; Baron-Cohen et al., 2000; Howard et al., 2000; Sparks et al., 2002; Schumann et al., 2004, 2009; Hazlett et al., 2009; Mosconi et al., 2009; Groen et al., 2010; Stigler et al., 2011; Nordahl et al., 2012). Studies relating amygdala volume to the degree of social impairment have had mixed results. Two studies found social impairment correlated with decreases in amygdala volume (Nacewicz et al., 2006; Mosconi et al., 2009), one with larger amygdala volume (Schumann et al., 2009), and two others no relationship at all (Dziobek et al., 2006; Juranek et al., 2006). The two studies that found positive correlation assessed social ability by experimental procedures, such as eye-tracking, rating joint attention from camera recordings, or facial emotion recognition tasks (Nacewicz et al., 2006; Mosconi et al., 2009). The other three studies which found no correlation or negative correlation (Dziobek et al., 2006; Juranek et al., 2006; Schumann et al., 2009) instead assessed social ability through clinical interviews such as the Autism Diagnostic Observation Schedule (ADOS) (Lord et al., 2000) or Autism Diagnostic Interview-Revised (ADI-R) (Lord et al., 1994), demonstrating the complexity of characterizing social ability and of relating clinical behaviors to results of cognitive tests. Other areas in which there has been some degree of correlation observed to measures of social function have included the medial PFC (Rojas et al., 2006; Schulte-Ruther et al., 2011), inferior frontal gyrus (Rojas et al., 2006), superior temporal sulcus (Pelphrey et al., 2005), FG (Greimel et al., 2010), temporal-parietal junction (TPJ) (Lombardo et al., 2011), and the anterior cingulate cortex (ACC) (Scott-Van Zeeland et al., 2010b).

### Language impairment

Delayed or atypical language development is a core feature of ASD (American Psychiatric Association, 2000). Language ability can vary enormously, from the 25% who never develop functional language to mild abnormalities in prosody (Mody et al., 2013). Neuroimaging studies of language function in autism have focused on language-associated brain regions such lateral inferior frontal cortex, including Broca’s area, and temporoparietal cortex, which contains Wernicke’s area (Shapleske et al., 1999; Dronkers et al., 2007). Language-associated areas are anatomically and functionally asymmetric, with predominance normally found in the hemisphere opposite the dominant hand. Although results are mixed, observations from multiple studies have suggested that asymmetry is often reduced or reversed in ASD (e.g., Herbert et al., 2002, 2005; Rojas et al., 2002, 2005; Just et al., 2004; McAlonan et al., 2005; Knaus et al., 2009; Anderson et al., 2010; Catarino et al., 2011).

Bigler et al. (2007) reported on gray matter volumes of the superior temporal gyrus in a group of 30 children with ASD compared to 39 controls matched on age and IQ; 13 of the controls had reading deficits. They found that superior temporal gyrus volume correlated with a standardized measure of language ability in the control group, but not the children with ASD. An early fMRI study reported that adults with ASD did not exhibit normal lateralization of brain response to vocal stimuli (Gervais et al., 2004). A more recent fMRI study of brain activity of sleeping toddlers (40 ASD and 40 matched controls, aged 12–48 months) during the reading of a bedtime story found that the ASD group did not show normal left-lateralized responses; there was instead a right-lateralized temporal cortex response which was most pronounced in the children toward the older end of the age range studied, suggesting an increasingly deviant developmental trajectory of laterality. Interestingly, within the ASD group, greater right hemisphere activity correlated with less severe symptoms (Redcay and Courchesne, 2008; Eyler et al., 2012).

These findings are consistent with those from a study comparing ASD and specific language impairment (SLI), a disorder in which delayed language development is present despite preservation of other cognitive abilities, and for which there is evidence of shared genetic risks with ASD (Fisher et al., 2003). This study compared children aged 6–12 years with SLI and typical controls to individuals with ASD with and without language impairment (De Fosse et al., 2004). They found overall larger brain volumes in the children with ASD. However, while reversed asymmetry of language-associated regions was present in the two groups that had language impairment, it was not present in the ASD group with normal language ability, suggesting that the asymmetry was more related to the presence or absence of language impairment than the ASD diagnosis.

### Restricted and repetitive interests and behaviors

The third core domain of ASD as defined in DSM-IV is the presence of restricted interests and RRIB, including both “higher order” RRIB, such as unusual preoccupations or patterns of interests and compulsive adherence to rituals or routines, and “lower order” RRIB, referring to stereotyped and repetitive motor mannerisms and preoccupation with parts of objects (Lord et al., 1994). Much of the work on RRIB has focused on the relationships between the frontal cortex and basal ganglia, taking as a model conditions with similar features such as obsessive-compulsive disorder (Scarone et al., 1992; Rosenberg et al., 1997) and Tourette syndrome (Peterson et al., 2003; Langen et al., 2012). Volumes of frontal regions have been correlated with RRB severity in ASD (Hardan et al., 2003; Rojas et al., 2006; Ecker et al., 2012), and in fMRI studies of tasks requiring cognitive inhibition, RRB severity has been shown to correlate with abnormal activation in areas including dorsolateral PFC, anterior cingulate, and the intraparietal cortex (Shafritz et al., 2008; Agam et al., 2010). Studies relating RRB symptoms to basal ganglia volumes have had mixed results. In a comparatively large sample of individuals with autism (*n* = 99; TD: *n* = 89), Langen et al. (2009) found that caudate volume was negatively correlated with insistence on sameness within higher order RRB. However, in Hardan et al. (2003), only scores on lower order RRB complex mannerisms were negatively correlated with caudate volume. Two other studies that included subjects across the autism spectrum and did not exclude ID found instead a positive correlation between caudate volume and severity of RRB (Hollander et al., 2005; Rojas et al., 2006).

### Sensory abnormalities

Abnormal sensory function, either hyper- or hyposensitivity, is extremely common in ASD. Described as an associated feature in DSM-IV-TR (American Psychiatric Association, 2000), in DSM-5 it was changed to be one of the diagnostic criteria in the restricted/RRIB category (American Psychiatric Association, 2013). Although key brain regions for sensory function such as the primary somatosensory cortex and insula are among those most consistently showing abnormalities in ASD, to date few neuroimaging studies have examined imaging correlates of abnormal sensory function in ASD directly. Cascio et al. (2012) used fMRI to compare responses to pleasant, neutral and unpleasant tactile stimuli between a sample of 13 adults with ASD and 14 matched controls. They found that the subjective descriptions of the sensations were on average similar between the groups, although there was more variability in the ASD responses, highlighting the heterogeneity within the group. Despite the similarity of the subjective reports, there were significant differences in brain activation: the ASD group had significantly less BOLD response to the pleasant and neutral stimuli, but some areas of increased activation during the unpleasant stimuli, including primary somatosensory cortex and insula. Increased activation in the insula correlated with social impairment scores, supporting the theory put forth by some that abnormal sensory function during early development may contribute to abnormal social development (Hilton et al., 2010). The thalamus has also been examined due to its central role in sensory processing. While thalamic volumes have not been found to relate to sensory function, a magnetic resonance spectroscopy study observed indications of a relationship between thalamic brain metabolites (*N*-acetyl aspartate and glutamate + glutamine) and sensory function (Hardan et al., 2008a,b). Another study reported that GM volume in the brainstem and oral sensitivity measures were associated in high-functioning ASD (Jou et al., 2009).

### Anxiety symptoms

Anxiety symptoms are also very common in ASD, estimated to affect over 40% (de Bruin et al., 2007; Eussen et al., 2012; Strang et al., 2012). A handful of studies have looked at how varying levels of anxiety affects neuroimaging findings in ASD, most of which have targeted structures in the limbic system. Higher levels of social anxiety (Corbett et al., 2009), and generalized anxiety/depression scores in children (Juranek et al., 2006) have each been correlated with decreased amygdala volume. fMRI studies have been more mixed. Kleinhans et al. (2010) found higher social anxiety scores correlated with reduced activation in the FG and greater activation in the amygdala in adults in response to angry and fearful faces. In contrast, another study in adolescents showed that brain activations were not associated with depression or anxiety scores (Weng et al., 2011).

### Poor attention and impulsivity

Symptoms such as inattention, hyperactivity, and impulsivity (American Psychiatric Association, 2000) are present in a majority of individuals with ASD (Schatz et al., 2002; Goldstein and Schwebach, 2004). The frequency of these types of symptoms in ASD was previously acknowledged through exclusion of a separate diagnosis of ADHD in the presence of PDD. Recognition that this exclusion unfortunately diminished the likelihood of recognition of co-occurring ADHD and associated symptoms resulted in its removal in DSM-5. Although work to date on ADHD and ASD has been largely been done separately, making explicit comparison between the two conditions and difficult, data thus far has demonstrated that there is significant overlap in both affected brain regions and genetic risk factors in the two disorders (Gargaro et al., 2011). This has led to hypotheses that that the underlying biology for both may fall along a continuum, with increasingly prominent social impairments as one moves from ADHD to ASD, and suggestions that more research studies should include both groups to allow direct comparisons (Brieber et al., 2007; Rommelse et al., 2011).

## Summary of Findings

Most studies in autism have not had understanding heterogeneity as a goal, but instead have effectively treated it as noise in the search for brain differences that correspond to the categorical diagnosis. These efforts have had some success. Findings have implicated many regions with prominent roles in social cognition, such as the superior temporal sulcus, amygdala, and insula (Di Martino et al., 2009). Volumetric and functional differences appear to be more pronounced in younger individuals, with a tendency toward larger volumes earlier in life (Courchesne et al., 2011; Wolff, 2013). With maturation these differences decrease in magnitude, particularly for brain structure, such that by adolescence summative measures such as total brain volume are not significantly different than controls. Other measures of brain structure continue to show differences into adulthood, and newer multivariate techniques have been able to identify subtle and widespread cortical differences consistent with the ongoing differences in function and behavior (Ecker et al., 2010). Abnormalities in structural and functional measures of connectivity are a consistent finding.

Efforts to address the heterogeneity of autism in neuroimaging studies have chiefly taken two forms: the first, to subdivide ASD into more homogeneous subgroups, using a variety of criteria; the second, to take a dimensional approach to examining the relationship between neuroimaging data and clinical features. Far fewer studies have been done using these approaches, and sample sizes are often quite modest; negative findings in particular may reflect lack of statistical power, or that studies using more sensitive measures have not yet been performed.

Clinically defined categories have included subgroups such as Aspergers’s versus narrowly defined Autism, ASD with and without significant language impairment, and low-functioning versus high-functioning autism. None of these comparisons have provided a strong case for a neurobiologically robust and distinct subtype, which is not to say variation along these clinical dimensions is not of ongoing interest. The relationship of ID to the pathophysiology of ASD has continued to be a challenging issue, complicated by the fact that brain imaging studies of individuals with significant ID are very difficult to carry out, and so samples including these subjects are often underpowered. This has created a situation where despite the predominance of ID in ASD, most imaging studies, particularly those with the sample sizes necessary for multivariate analyses, are carried out in ASD individuals with normal or near-normal IQ.

There have been a few specific risk factors identified with strong enough associations to ASD to look at affected individuals as specific subgroups. One of these is male gender, whose much higher rates compared to females imply the presence of risk factors unique to males. The few structural imaging studies explicitly designed for gender comparison have generally not found significant differences in the pattern of abnormalities, except for the likelihood of females to show more pronounced brain differences than males. Of other specific genetic risk factors, by far the most work has been done in FXS. Here, the pattern of imaging findings appeared to be driven by the FXS genotype, regardless of whether the individuals met criteria for ASD or not. Some of the brain differences associated with the presence of autistic features in the FXS and non-FXS groups affected similar regions but in opposite directions.

The other most frequently used approach in neuroimaging to heterogeneity with ASD has been through relating dimensional variation in clinical or cognitive measures to brain measures. Although these analyses have been generally intended less to describe heterogeneity than to strengthen the case for the likely relevance of the observed brain differences to the clinical symptoms, they can be informative about whether the variation observed clinically and in imaging measures are likely to be reflective of each other. Although reported findings have been mixed, many have been consistent with what would be expected for brain regions known to be associated with specific functions, as described above.

## Conclusion

So, what has neuroimaging told us about heterogeneity in ASD? The main finding may be that neuroimaging provides no refuge from the multiplicity of presentations and candidate risk factors found in the clinic and the genetics laboratory. Studies to date have made progress in identifying patterns of brain abnormalities present in groups of people with ASD, but inconsistency between study results is still more the norm than the exception, and biomarkers robust enough to be meaningful on an individual level have yet to be identified. Adoption of multivariate methods and pattern identification methods based on techniques such as machine learning may improve results, as more reflective of the widespread and subtle morphologic differences that have become apparent with larger scale studies (Ecker et al., 2012), and are consistent with current hypotheses of ASD as being rooted in abnormalities of synaptic development (State and Sestan, 2012). The difficulty with this approach, however, as has been discussed extensively elsewhere (Hyman, 2010), is that it continues to center around a concept of autism, or even the broader range of ASD, as a discrete entity. Such a designation can be highly useful from the practical level of providing a diagnostic label and indications for intervention. However, as a constraint for inquiries into biology, it may more distort than illuminate. Autistic disorders exist not only as a spectrum within the realm of pathology, but also the severe end of a set of continuous traits which extend into the general population, and do not have clear boundaries with other disorders such as ADHD (Lai et al., 2013b). Abundant evidence from epidemiologic, genetic and twin studies supports the common nature of the risk factors affecting autistic traits within individuals meeting criteria for the disorder and in the general population (Robinson et al., 2011; Ronald and Hoekstra, 2011; Lundstrom et al., 2012). ASD might be better considered a name assigned to designate individuals whose expression of a particular set of continuously varying traits has reached a certain threshold of severity (Uher and Rutter, 2012).

These issues have been much discussed of late, prompted by the most recent revision of DSM (Kendler, 2012; Lord and Jones, 2012). Although DSM-5 left the previous categorical system largely intact, acknowledging its clinical utility and the lack of sufficient evidence to support more substantive revision, for research purposes there has been increasing support for decreasing the emphasis on categorical diagnoses (Uher and Rutter, 2012). Proposed alternatives include transdiagnostic dimensional approaches such as the Research Domain Criteria (RDoCs) currently under development at the National Institutes of Mental Health (NIMH) in the U.S.^2^ (Insel et al., 2010), which focus on simpler constructs (for example, response to social stimuli, or working memory) that may be more amenable to linking across multiple levels of neural, cognitive, and behavioral function regardless of which clinical syndrome a particular feature is occurring within.

Neuroimaging has revolutionized our understanding of neurodevelopmental disorders by affording observations of brain structure and function *in vivo*, including the tracing of developmental trajectories in children and adolescents from the very first months of life. It should be kept in mind that despite the rapid technical advances in the field, MRI techniques remain limited to a level of spatial and temporal resolution too coarse to visualize the synaptic or neuronal-level abnormalities that may be core features of disorders such as ASD. If this is the level from which heterogeneity arises, neuroimaging may ultimately not be the best tool for parsing these differences. However, in combination with more finely grained methods such as post-mortem tissue analysis and animal models, neuroimaging studies have the potential to provide a critical intermediate step between risk factors such as specific genes and the cognitive or behavioral features of interest. Such approaches, by focusing on the links between genetic, biological, and behavioral domains, allow us the opportunity to deconstruct our conceptions of ASD back to where they can be grounded in biology. Eventually, heterogeneity may no longer be considered as noise in neuroimaging studies of ASD, and instead take its place as a guide to pathophysiology (Brock, 2011; Georgiades et al., 2013).

## Conflict of Interest Statement

The authors declare that the research was conducted in the absence of any commercial or financial relationships that could be construed as a potential conflict of interest.

## Footnotes

^1^http://fcon_1000.projects.nitrc.org/indi/abide/

^2^www.nimh.nih.gov/research-priorities/rdoc
